# Supplementary material for: A Humanized Yeast Phenomic Model of Deoxycytidine Kinase to Predict Genetic Buffering of Nucleoside Analog Cytotoxicity
Source: Genes (Basel). 2019 Sep 30;10(10):770. doi: 10.3390/genes10100770 (PMC6826991; doi:10.3390/genes10100770)
Supplement: Supplementary file 1 [file genes-10-00770-s001.zip › Additional_Files/Additional_File7_GOTermHeatmaps.zip!/Additional_File7_GOTermHeatmaps/sister_chromatid_segregation.pdf]

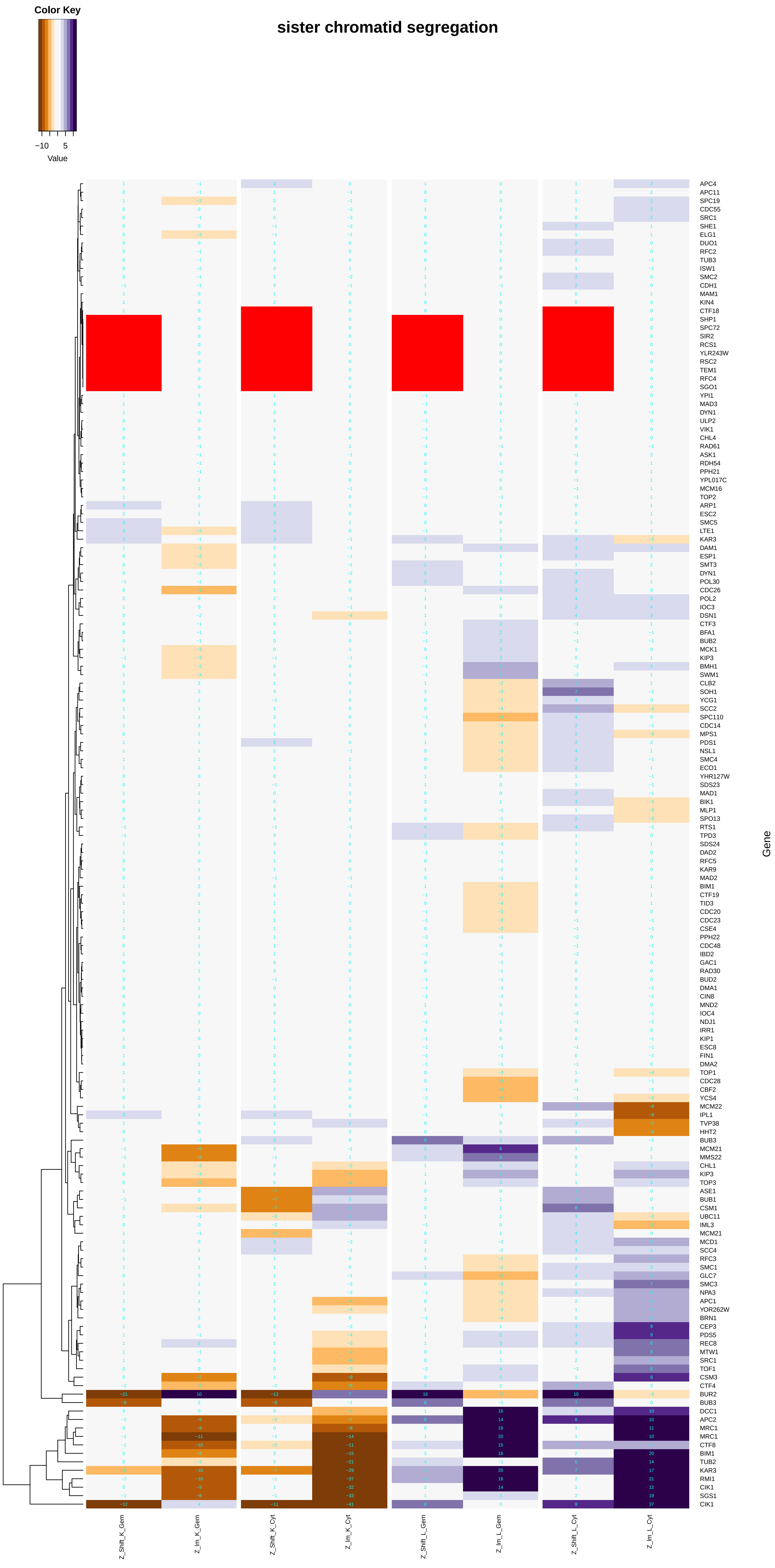

Color Key
−10 5
Value
sister chromatid segregation
Gene
Z_Shift_K_Gem
Z_Im_K_Gem
Z_Shift_K_Cyt
Z_Im_K_Cyt
Z_Shift_L_Gem
Z_Im_L_Gem
Z_Shift_L_Cyt
Z_Im_L_Cyt
APC4
APC11
SPC19
CDC55
SRC1
SHE1
ELG1
DUO1
RFC2
TUB3
ISW1
SMC2
CDH1
MAM1
KIN4
CTF18
SHP1
SPC72
SIR2
RCS1
YLR243W
RSC2
TEM1
RFC4
SGO1
YPI1
MAD3
DYN1
ULP2
VIK1
CHL4
RAD61
ASK1
RDH54
PPH21
YPL017C
MCM16
TOP2
ARP1
ESC2
SMC5
LTE1
KAR3
DAM1
ESP1
SMT3
DYN1
POL30
CDC26
POL2
IOC3
DSN1
CTF3
BFA1
BUB2
MCK1
KIP3
BMH1
SWM1
CLB2
SOH1
YCG1
SCC2
SPC110
CDC14
MPS1
PDS1
NSL1
SMC4
ECO1
YHR127W
SDS23
MAD1
BIK1
MLP1
SPO13
RTS1
TPD3
SDS24
DAD2
RFC5
KAR9
MAD2
BIM1
CTF19
TID3
CDC20
CDC23
CSE4
PPH22
CDC48
IBD2
GAC1
RAD30
BUD2
DMA1
CIN8
MND2
IOC4
NDJ1
IRR1
KIP1
ESC8
FIN1
DMA2
TOP1
CDC28
CBF2
YCS4
MCM22
IPL1
TVP38
HHT2
BUB3
MCM21
MMS22
CHL1
KIP3
TOP3
ASE1
BUB1
CSM1
UBC11
IML3
MCM21
MCD1
SCC4
RFC3
SMC1
GLC7
SMC3
NPA3
APC1
YOR262W
BRN1
CEP3
PDS5
REC8
MTW1
SRC1
TOF1
CSM3
CTF4
BUR2
BUB3
DCC1
APC2
MRC1
MRC1
CTF8
BIM1
TUB2
KAR3
RMI1
CIK1
SGS1
CIK1

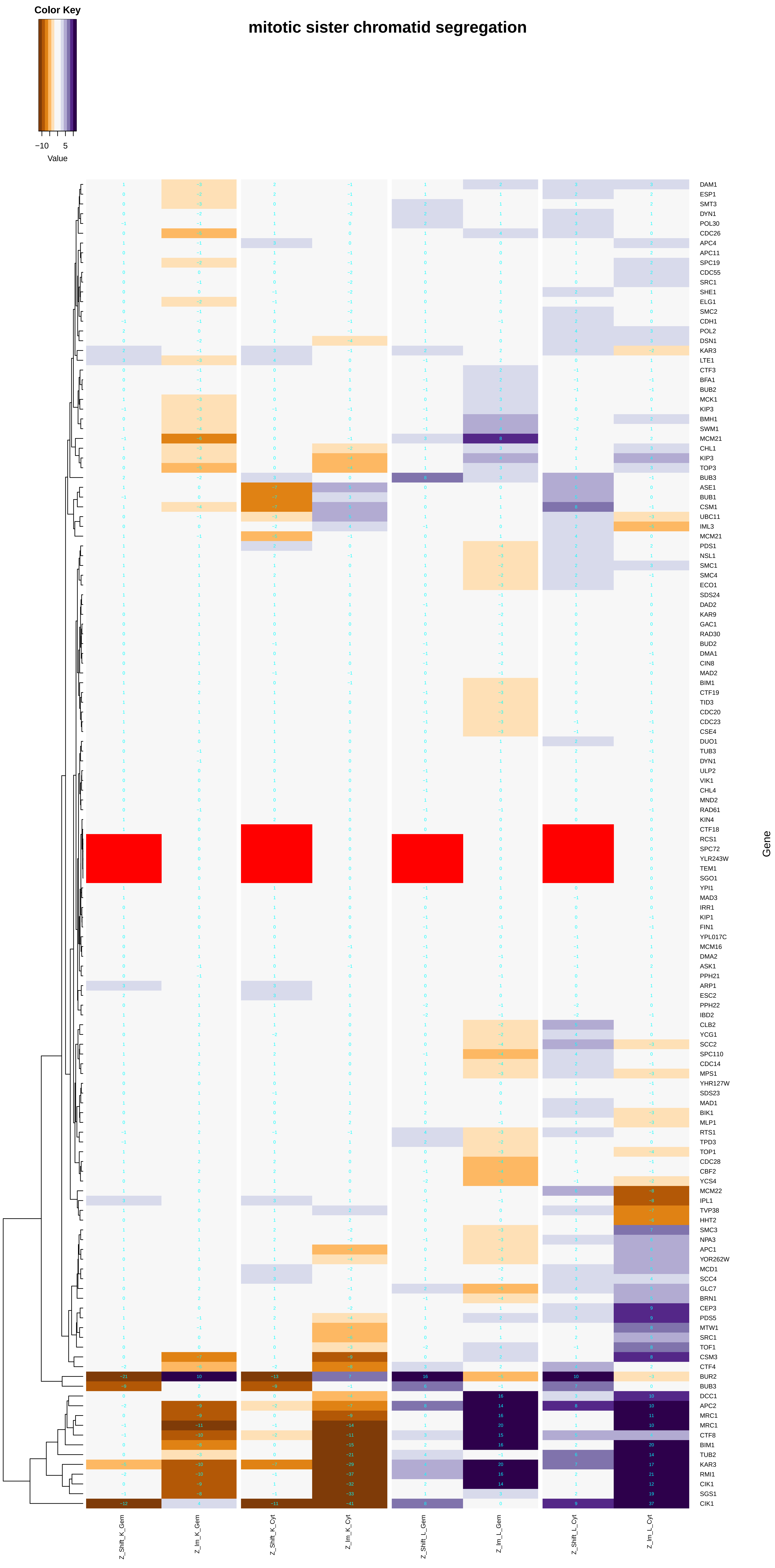
mitotic sister chromatid segregation
Color Key
−10
5
Value
Gene
Z_Shift_K_Gem
Z_lm_K_Gem
Z_Shift_K_Cyt
Z_lm_K_Cyt
Z_Shift_L_Gem
Z_lm_L_Gem
Z_Shift_L_Cyt
Z_lm_L_Cyt
DAM1
ESP1
SMT3
DYN1
POL30
CDC26
APC4
APC11
SPC19
CDC55
SRC1
SHE1
ELG1
SMC2
CDH1
POL2
DSN1
KAR3
LTE1
CTF3
BFA1
BUB2
MCK1
KIP3
BMH1
SWM1
MCM21
CHL1
KIP3
TOP3
BUB3
ASE1
BUB1
CSM1
UBC11
IML3
MCM21
PDS1
NSL1
SMC1
SMC4
ECO1
SDS24
DAD2
KAR9
GAC1
RAD30
BUD2
DMA1
CIN8
MAD2
BIM1
CTF19
TID3
CDC20
CDC23
CSE4
DUO1
TUB3
DYN1
ULP2
VIK1
CHL4
MND2
RAD61
KIN4
CTF18
RCS1
SPC72
YLR243W
TEM1
SGO1
YPI1
MAD3
IRR1
KIP1
FIN1
YPL017C
MCM16
DMA2
ASK1
PPH21
ARP1
ESC2
PPH22
IBD2
CLB2
YCG1
SCC2
SPC110
CDC14
MPS1
YHR127W
SDS23
MAD1
BIK1
MLP1
RTS1
TPD3
TOP1
CDC28
CBF2
YCS4
MCM22
IPL1
TVP38
HHT2
SMC3
NPA3
APC1
YOR262W
MCD1
SCC4
GLC7
BRN1
CEP3
PDS5
MTW1
SRC1
TOF1
CSM3
CTF4
BUR2
BUB3
DCC1
APC2
MRC1
MRC1
CTF8
BIM1
TUB2
KAR3
RMI1
CIK1
SGS1
CIK1

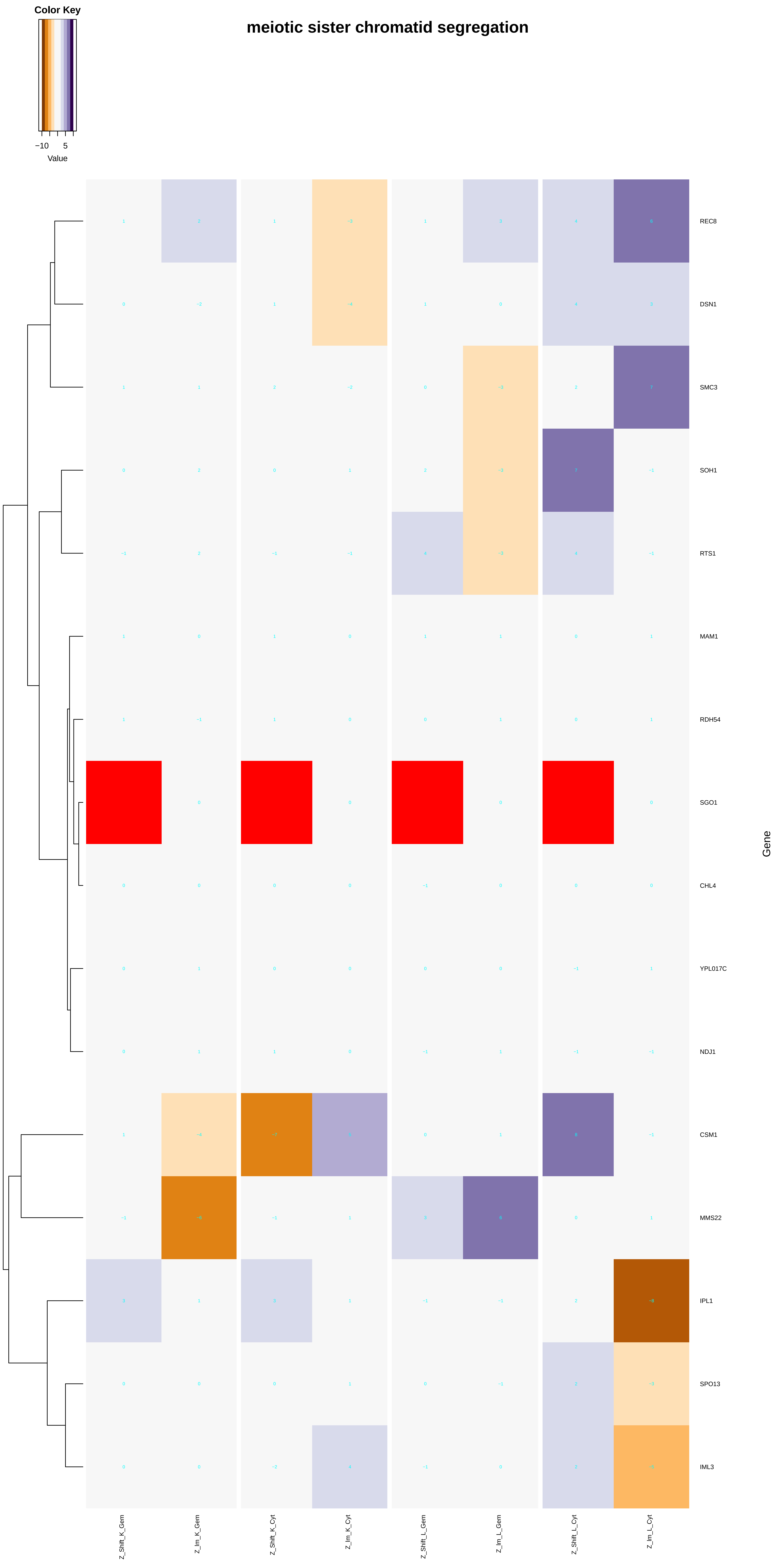

Color Key
−10
5
Value
meiotic sister chromatid segregation
REC8
DSN1
SMC3
SOH1
RTS1
MAM1
RDH54
SGO1
CHL4
YPL017C
NDJ1
CSM1
MMS22
IPL1
SPO13
IML3
Gene
Z_Shift_K_Gem
Z_Im_K_Gem
Z_Shift_K_Cyt
Z_Im_K_Cyt
Z_Shift_L_Gem
Z_Im_L_Gem
Z_Shift_L_Cyt
Z_Im_L_Cyt
